# T211. BASIC SELF-DISTURBANCE AS A PREDICTOR OF DETERIORATION IN ATTENUATED PSYCHOSIS: A 1-YEAR FOLLOW-UP STUDY AMONG COMMUNITY-DWELLING ADOLESCENTS

**DOI:** 10.1093/schbul/sby016.487

**Published:** 2018-04-01

**Authors:** Dan Koren, Liza Lacoua, Lily Rothshield, Maya Rothbaum, Neta Cohen, Josef Parnas

**Affiliations:** 1 University of Haifa; 2 University of Copenhagen

## Abstract

**Background:**

Phenomenological research indicates that disturbance of the basic sense of self may be a core phenotypic marker of schizophrenia spectrum disorders. Basic self-disturbance (SD) refers to a disruption of the sense of first-person perspective and self-presence that is associated with a variety of anomalous subjective experiences. Recent studies including from our group provided first, preliminary support for the notion that SD is related to attenuated psychosis symptoms (APS) and depression among clinical (i.e., treatment-seeking) and non-clinical samples of non-psychotic adolescents. However, very few studies, if any at all, have looked at the ability of SD to predict change in APS and depression over time. The goal of this study was to address this lacuna in the literature by examining the unique and added contribution of SD to the prediction of change over time in APS and depression among community-dwelling adolescents.

**Methods:**

The 1-year longitudinal relationship between SD and change in APS and depression were explored in a sample of 100 non-help-seeking adolescents (age 13–15) from the community. SD was assessed with the Examination of Anomalous Self-Experience (EASE), prodromal symptoms and syndromes were assessed with the Structured Interview for Prodromal Syndromes (SIPS), present and lifetime diagnoses of schizophrenia-spectrum and depression disorders were assessed with the Kiddie Schedule for Affective Disorders and Schizophrenia (K-SADS), level of distress with the Mood and Anxiety States Questionnaire (MASQ), and psychosocial functioning with the with the Strength and Difficulties Questionnaire (SDQ).

**Results:**

Seventy-seven (77%) adolescents out of the 100 that had been assessed at baseline were available and agreed to participate in the 1-year follow-up (Mean=1.4, S.D.=0.8). Except for a diagnosis of an affective disorder, which was slightly less prevalent among those who returned for the follow-up assessment, there were no significant differences between those who were available and those who lost for the follow-up assessment on any of the major socio-demographic or clinical variables at baseline. Consistent with our first hypothesis, SD at baseline predicted a significant amount of variance in APS change over time (R-squared=0.10, F= 8.61, p=0.004). However, inconsistent with our second hypothesis, SD at baseline did not have a significant added contribution to the prediction of APS change when APS at baseline was controlled for (R-squared difference=0.02, F=1.83, p=0.18).

**Discussion:**

These results provide preliminary support for a prospective association between SD and deterioration in prodromal symptoms among adolescents from the community. However, they fail to support an added value of SD over and above baseline APS for the prediction of APS deterioration. Because SD was assessed only at baseline, they leave unanswered the degree to which change in SD is associated with a change in APS and depression.

